# Parental occupational exposure to endocrine disrupting chemicals and male genital malformations: A study in the danish national birth cohort study

**DOI:** 10.1186/1476-069X-10-3

**Published:** 2011-01-14

**Authors:** María M Morales-Suárez-Varela, Gunnar V Toft, Morten S Jensen, Cecilia Ramlau-Hansen, Linda Kaerlev, Ane-Marie Thulstrup, Agustín Llopis-González, Jørn Olsen, Jens P Bonde

**Affiliations:** 1 Unit of Public Health and Environmental Care, Department of Preventive Medicine, University of Valencia. Valencia, Spain; 2 CIBER Epidemiology and Public Health (CIBERESP), Spain; 3 Center for Public Health Research (CSISP), Valencia, Spain; 4 Department of Occupational Medicine, Aarhus University Hospital, Denmark; 5 Perinatal Epidemiology Research Unit, Departments of Obstetrics and Paediatrics, Aarhus University Hospital, Skejby, Denmark; 6 Center for National Clinical Databases South, Department of Research and HTA, Odense University Hospital, Denmark; 7 Department of Epidemiology, School of Public Health, UCLA, Los Angeles, USA; 8 Department of Occupational and Environmental Medicine, Bispebjerg Hospital, Denmark; 9 Institute of Public Health, University of Copenhagen, Denmark

## Abstract

**Background:**

Sex hormones closely regulate development of the male genital organs during fetal life. The hypothesis that xenobiotics may disrupt endogenous hormonal signalling has received considerable scientific attention, but human evidence is scarce.

**Objectives:**

We analyse occurrence of hypospadias and cryptorchidism according to maternal and paternal occupational exposure to possible endocrine disrupting chemicals.

**Methods:**

We conducted a follow-up study of 45,341 male singleton deliveries in the Danish National Birth Cohort during 1997-2009. Information on work during pregnancy was obtained by telephone interviews around gestational week 16. Parents' job titles were classified according to DISCO-88. A job exposure matrix for endocrine disrupting chemicals (EDCs) was implemented to assess occupational exposures. The Medical Birth and National Hospital Register provided data on congenital anomalies diagnosed at birth or during follow-up, which ended in 2009. Crude and adjusted hazard ratios (HR) were obtained from Cox regression models.

**Results:**

Among all pregnancies, 6.3% were classified as possibly or probably exposed to EDCs. The most prevalent occupations conferring possible exposure were cleaners, laboratory technicians, hairdressers and agricultural workers (58% of all potentially exposed). The final cumulative incidence of cryptorchidism in boys was 2.2% (1002 cases), and of hypospadias 0.6% (262 cases). The occurrence of hypospadias increased when mothers were probably [HRa = 1.8 (95% CI 1.0-2.6)] or possibly exposed to one or more EDCs [HRa = 2.6 (95% CI 1.8-3.4). Possible paternal exposure to heavy metals increased the risk of hypospadias [HRa 2.2 (95% CI: 1.0-3.4)] and cryptorchidism [HRa 1.9 (95% CI: 1.1-2.7)]. None of the exposure groups reached statistical significance.

**Conclusion:**

The study provides some but limited evidence that occupational exposure to possible endocrine disrupting chemicals during pregnancy increases the risk of hypospadias.

## Background

Cryptorchidism (incomplete testicular descent) is a common congenital disorder, but may also be acquired [1]. The prevalence at three months of age was 1.9% in a Danish sample [2], 1.0 in a sample from USA [3] and between 1.6% [4] and 2.4% [5] in large samples from UK. Hypospadias (abnormal location of the urethral orifice) is observed in 2-4 per 1,000 male births in Europe [6,7]. There are indications that the prevalence of cryptorchidism and hypospadias increased from the 60s to the 80s in Europe and in the USA, although data providing this information have important methodological limitations [8-10].

The fetal development of the male reproductive organs is controlled by sex hormones, and in particular androgens play a crucial role during the first trimester of pregnancy [11]. Fetal exposure to chemicals with anti-androgen or estrogen-like activity may interfere with normal hormonal signalling, which may increase the risk of cryptorchidism, hypospadias and other male reproductive disorders [12,13].

Many widespread chemicals including dioxins and furans, polychlorinated biphenyls, organochlorine pesticides, phthalate esters, brominated flame-retardants and some heavy metals have been identified as possible endocrine disrupters [14]. Therefore, it remains an important issue to corroborate or refute the hypothesis role of these chemicals in male reproductive disorders [15-23]. In this study, we examine occupational exposure to potentially endocrine disrupting compounds, utilizing data from a large Danish National Birth Cohort. The objective was to estimate the risk of hypospadias and cryptorchidism according to maternal and paternal occupational exposures to chemicals interfering with hormonal homeostasis with specific focus on fetal exposure during pregnancy.

## Methods

### Study population

We used data from the Danish National Birth Cohort (DNBC), which is a nationwide study among pregnant women and their offspring [24]. Between March 1997 and November 2002 pregnant women across Denmark were informed about the study during their first antenatal visit to the general practitioner. About 60% of invited women accepted the invitation by signing an informed consent form [25]. The only exclusion criteria were not having access to a telephone, not speaking Danish well enough to complete the interview, and not intending to carry the pregnancy to term.

A total of 101,052 pregnant women were enrolled in the study, and 92,892 participated in the first interview at approximately 16 weeks of gestation (interquartile range, 11-25). Women were contacted by trained female telephone interviewers. Interviews were classified as missing if women were not reached at the scheduled time or after three additional attempts to make contact. Interviews were cancelled if the contacted woman was no longer pregnant. All Regional Science Ethics Committees in Denmark have approved the DNBC and before we initiated this study we obtained approval from the Danish Data Protection Agency.

### Exposure assessment

In the first study interview, mothers answered questions about their work three months before pregnancy and during pregnancy, and also about the father's work. Mothers provided information on their current or most recent jobs and about the father's job. This information was coded according to the Danish version of International Standard Classification of Occupation (DISCO-88), which contains 348 job titles [26].

Maternal and paternal occupational codes were classified into categories of potential exposure to possible endocrine disrupting chemicals (EDCs), using a job exposure matrix that was developed by a Dutch-British group [14]. Three occupational exposure experts classified independently all the DISCO-88 job titles into three exposure categories: "unlikely", "possible" or "probable" exposure to one or more of seven groups of EDCs: pesticides, organochlorine compounds, phthalate esters, alkyl phenols, bis-phenols, heavy metals (cadmium, lead, mercury), and other compounds (hormone disrupting chemicals). The classification was scored according to the following criteria: (0) Exposure among the workers with this job title is very "unlikely" (1) There is a "possibility" that some of the workers with this job title are exposed (but the probability is fairly low) (2) There is some "probability" that at least a proportion of the workers with this job title are somewhat exposed. The job exposure ratings of the three experts were compared. If there was disagreement exceeding one, discussion to reach consensus was performed but differences of one category were allowed. The final code assigned each occupation was the median value of the three expert scores. Two-step analyses were made of firstly: combined EDC exposure, and secondly: to each of the chemical groups.

### Ascertainment of outcome

Pregnancy outcome was identified and assessed using Danish national registers, by using the unique identifier code given to each individual at birth. The Medical Birth Register and the Civil Registration System were used to obtain data on live births, stillbirths and emigration of the mother before pregnancy ended. Other pregnancy outcomes were identified in the National Hospital Discharge Register during the period 1997 through 2009. Thus, the youngest child was 6 years old and the oldest was 13 years old at the end of follow-up. Less than one percent of the study pregnancies could not be linked to registry data, in which case information from the pregnancy interview was used instead.

The National Hospital Discharge Register included information about congenital anomalies based on the 10^th ^Revision of the International Classification of Diseases (ICD10 codes DQ00-DQ99). The registry covered 100% of Denmark's hospitals during the study period including all inpatient and outpatient clinic diagnoses and surgeries performed. We identified 262 cases of hypospadias (ICD10 codes DQ54-DQ549, with the exeption of DQ544) and 1002 cases of cryptorchidism (ICD10 codes Q53, Q531, Q531A, Q532, Q532A, and Q539). Twenty-two boys had both anomalies. Information on surgical correction of cryptorchidism, orchiopexy (codes KKFH00, KKFH01, and KKFH10 in the Nordic Classification of Surgical Procedures) was included in the analyses. Orchiopexy indicates that the disease (cryptorchidism) is persisting and requires surgery.

### Statistical analysis

A Cox regression analysis with the boys' age as the time variable was performed to compare the occurrence of hypospadias and cryptorchidism in the "possible" and "probable" exposure categories using the baseline "unlikely" exposure category as reference. The boys entered the risk set at birth and were followed until their age at first diagnosis, surgery, death, emigration from Denmark, or end of follow up (October 21, 2009), whichever came first. Separate analyses were made for cryptorchidism with and without orchiopexy. We present the estimated HRs with 95% confidence intervals (95% CI). The following fixed set of covariates was defined a priori and included in the models regardless of their effect on the risk estimates: Age of the mother and the father in first trimester (≤20, 20.1-30, 30.1-35, 35.1-40 and over 40.1 years); mothers' pre-pregnancy body mass index (kg/m^2^) according to three body mass index categories (< 20, 20-30 and > 30); Previous spontaneous abortion (yes/no); parity (primiparous (1), 2-3, more than 4); birth weight of boys (<2500 g, 2500-4000 g and >4000 g); gestational age (< 24 weeks, 24.1-32 weeks, 32.1 -37 weeks and ≥37.1 weeks) oral contraceptives used in the past (yes/no); treatment of infertility (yes/no); time to pregnancy (unexpected pregnancy, immediately, 1-2 months, 3-5 months, 6-12 months, ≥ 13 months); mother's alcohol consumption during pregnancy (yes/no); binge drinking defined as intake of at least five drinks at one occasion at least one time (yes/no); maternal smoking during pregnancy (no smoking, ≤10 cigarettes/day, 11-19 cigarettes/day, and ≥ 20 cigarettes/day); paternal smoking (no, yes-not every day, yes-every day), we considered: one cigarette = one cigarette equivalent, one cherrot = two cigarettes equivalent, one cigar = two cigarettes equivalent, one pipe = one cigarette and a half equivalent; gynecological disease (yes/no); maternal vegetarian diet (yes/no). The mothers' job titles were used to define their socio-occupational status. Women with a higher level of education (4 years beyond high school), or who worked in management positions, were classified as "high socio-occupational status". The "medium" category included skilled workers and women with medium-ranged training/level of education, while the "low" category included unskilled workers. All the analyses were performed using the SPSS software (version 14.0; SPSS Inc. Chicago III).

## Results

Of the 92,892 women participating in the first telephone interview, we excluded all subsequent pregnancies (n = 2,425), women whose pregnancies ended with induced abortion (n = 93), hydatidiform mole (n = 42), ectopic pregnancy (n = 24), multiple birth with no live born infants (n = 3), women who died during pregnancy (n = 1), and unknown outcomes of the pregnancy (n = 9). Women who did not work during pregnancy (n = 1,431) were also excluded. From the remaining 88,864 live-born singletons, 45,341 boys were available for analysiswith information about EDC exposure and follow-up time. Characteristics of the study population are presented in Table 1, and show that low socio-occupational status (SES) is associated with the risk of being exposed to ECDs. Other characteristics seemed to occur at similar proportions in the various EDC exposure categories.

**Table 1:** Characteristics of 45,341 pregnancies according to maternal occupational exposure to ECDs.

			Unlikely EDC exposure (N = 42,474)		Possible/Probably EDC exposure (N = 2,867)	
			**N**	**%**	**N**	**%**

**Mother**	**Age (years)**	≤ 20	335	0.8	39	1.4
		
		20.1-30	21606	50.9	1579	55.1
		
		30.1-35	15009	35.4	919	32.1
		
		35.1-40	4982	11.7	304	10.6
		
		≥ 40.1	525	1.2	26	0.9
	
	**Pre-pregnancy body mass index (kg/m**^**2**^**)**	BMI < 20.0	9874	24.0	700	24.5
		
		BMI 20.1 -30.0	22566	55.0	1502	52.6
		
		BMI > 30.1	8617	21.0	656	23.0
	
	**Socio-occupational status**	High	22105	53.9	954	34.5
		
		Medium	15574	38.0	1001	36.2
		
		Low	3352	8.2	812	29.3
	
	**Vegetarian diet**	Yes	478	1.3	15	0.6
	
	**Consuming alcohol during pregnancy**	Yes	2310	5.5	156	5.6
	
	**Binge drinking during pregnancy**	Yes	1845	4.4	132	4.7
	
	**Smoking during pregnancy (cigarettes/day)**	0	32944	80.0	2159	77.9
		
		≤10	5796	14.1	399	14.4
		
		10.1-19.9	1717	4.2	152	5.5
		
		≥20	728	1.8	61	2.2
	
	**History of spontaneous abortions**	Yes	7400	18.0	455	16.4
	
	**Parity**	1*	38426	90.5	2641	92.1
		
		≥2-3	4048	9.6	226	7.9
	
	**Oral contraceptives used in the past**	Yes	9990	24.3	678	24.5
	
	**Treated for infertility**	Yes	2310	5.5	156	5.6
	
	**Time to pregnancy**	Unplanned	8282	22.8	504	20.8
		
		Immediately	1575	4.3	94	3.9
		
		1 - 2.9 months	8645	23.8	555	22.9
		
		3 - 5.9 months	7113	19.6	473	19.5
		
		6 - 12 months	5558	15.3	401	16.6
		
		> 12.1 months	5089	14.0	395	16.3
	
	**Gynecologic diseases**	Yes	10598	25.8	650	23.5

**Father**	**Age (years)**	≤20	335	0.8	39	1.4
		
		20.1-30	21606	50.9	1579	55.1
		
		30.1-35	15011	35.4	919	32.1
		
		35.1-40	4982	11.7	304	10.6
		
		≥ 40.1	526	1.2	26	0.9
	
	**Smoking habit**	No	28000	69.4	1784	65.4
		
		Yes - less than daily	1269	3.1	67	2.5
		
		Yes - daily	11058	27.4	875	32.1

**Boys**	**Duration of gestation**	≤24 weeks	13	0.1	1	0.1
		
		24.1-32 weeks	284	0.7	22	0.8
		
		32.1 - 37 weeks	1954	4.9	152	5.7
		
		≥37.1 weeks	37350	94.3	2510	93.5
	
	**Birth weight**	<2500 g	1303	3.2	91	3.3
		
		2500.1-4000 g	30002	72.7	2071	74.4
		
		>4000.1 g	9982	24.2	620	22.3
	
	**Age of Cryptorchidism Diagnosis**	0 - 2 months	7	0.8	-	-
		
		2.1 - 24 months	204	23.3	10	18.9
		
		24.1 - 48 months	328	37.4	23	43.4
		
		≥48.1 months	337	38.5	20	37.7
	
	**Age of Hypospadias Diagnosis**	0 - 2 months	-	-	-	-
		
		2.1 - 24 months	6	2.8	-	-
		
		24.1 - 48 months	8	3.7	-	-
		
		≥ 48.1 months	202	93.5	28	100

The Danish National Birth Cohort (1997-2009), (N = 45,341).

Primiparous, including this birth.

Numbers may not add up because of missing data.

The cumulative incidence of cryptorchidism at the end of follow-up was 2.2% (1002 cases) and of hypospadias it was 0.6% (262 cases). Almost 0.6% of all women were classified as being possibly exposed to at least one of the seven classes of EDC's, while 5.7% were classified as being probably exposed. The most prevalent occupations conferring likely exposure to EDC's among women were cleaners, laboratory technicians, hairdressers and agricultural workers (58.0% of all the exposed women). Among fathers, 14.9% were classified as being possibly exposed to one or more classes of EDCs, while 11.9% fell into the probably exposed category.

**Figure 1:**
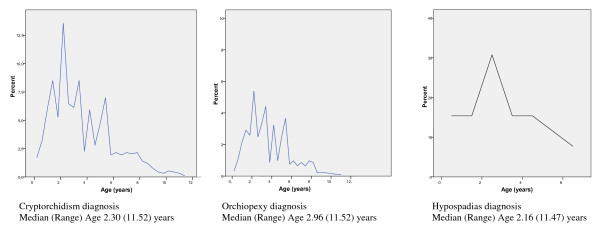
**Age distribution of first hospital referral for cryptorchisdism, orchiopexy and hypospadiasin the DNBC (1997-2009) **.

The age distributions for first hospital referral for cryptorchidism, orchiopexi and hypospadias are presented in Figure 1. The figures show the age when cases were diagnosed in the health care system and not the age when the anomalies became clinically manifest.

The risk of hypospadias but not of cryptorchidism was significantly elevated following possible and probable maternal exposure to one or more classes of EDCs (Table 2). The most prevalent occupations conferring possible exposure were cleaners, laboratory technicians, hairdressers and agricultural workers (58% of all potentially exposed). The increased risk of hypospadias was mostly related to potential exposure to pesticides, phthalate esters, alkyl phenols, bis-phenols and other compounds, although the confidence intervals for all the subgroup analyses were wide. Paternal exposure were not consistently related to increased risk of hypospadias, but an association with probable exposure to heavy metals [HRa 2.2 (95% CI: 1.0-3.4)] was observed. Cryptorchidism was associated with fathers' probable exposure to heavy metals [HRa 1.9 (95% CI: 1.1-2.7)] but not with fathers' possible exposure or with maternal exposure.

**Table 2:** Hazard ratios (HR) for hypospadias and cryptorchidism in boys according to prenatal exposure to endocrine disrupting chemicals conferred by parental occupational exposure (n = 45,341).

	EDCs occupational exposures	Mother's occupational exposure							Father's occupational exposure						
		
		Study population (n = 45341) (%)	Hypospadias (n = 262)			Cryptorchidism (n = 1002)			Study population (n = 45341) (%)	Hypospadias (n = 262)			Cryptorchidism (n = 1002)		
			
			N (CIP)	HRc	HRa (95% CI)	N (CIP)	HRc	HRa (95% CI)		N (CIP)	HRc	HRa (95% CI)	N (CIP)	HRc	HRa (95% CI)
**EDC** **exposure**	Unlikely	42474 (93.7)	876 (2.1)	1.0	1.0	876 (2.1)	1.0	1.0	38726 (85.4)	209 (0.5)	1.0	1.0	799 (2.1)	1.0	1.0
	
	Possible	277 (0.6)	7 (2.5)	2.4	2.6* (1.8-3.4)	7 (2.5)	1.2	1.6 (0.4-2.8)	410 (0.9)	-	-	-	7 (1.7)	0.9	0.8 (0.4-1.2)
	
	Probable	2590 (5.7)	46 (1.8)	1.4	1.8* (1.0-2.6)	46 (1.8)	0.9	0.8 (0.4-1.2)	6205 (13.7)	35 (0.6)	1.0	1.3 (0.7-1.9)	123 (2.0)	1.0	1.0 (0.8-1.4)

**Pesticides**	Unlikely	45030 (99.3)	242 (0.5)	1.0	1.0	927 (2.1)	1.0	1.0	41922 (92.5)	227 (0.5)	1.0	1.0	867 (2.1)	1.0	1.0
	
	Possible	62 (0.1)	1 (1.6)	3.0	1.2 (0.2-2.2)	-	-	-	1943 (4.3)	9 (0.5)	1.0	0.9 (0.3-1.5)	40 (2.1)	1.0	1.0 (0.7-1.3)
	
	Probable	249 (0.5)	1 (0.4)	0.8	1.1 (0.9-1.3)	2 (0.8)	0.6	0.5 (0.1-0.9)	1476 (3.3)	8 (0.5)	0.9	1.6 (0.8-2.4)	22 (1.5)	0.7	0.5 (0.3-0.7)

**Organochlorine** **compounds**	Unlikely	45104 (99.5)	243 (0.5)	1.0	1.0	923 (2.0)	1.0	1.0	43507 (96.0)	236 (0.5)	1.0	1.0	889 (2.0)	1.0	1.0
	
	Possible	214 (0.5)	1 (0.5)	0.9	1.6 (0.3-2.9)	6 (2.8)	1.2	0.7 (0.2-1.2)	782 (1.7)	4 (0.5)	1.0	1.2 (0.4-2.0)	19 (2.4)	1.2	0.9 (0.3-1.5)
	
	Probable	23 (0.1)	-	-	-	-	-	-	1052 (2.3)	4 (0.4)	0.7	1.1 (0.4-1.8)	21 (2.0)	1.0	1.0 (0.6-1.4)

**Phthalate** **esters**	Unlikely	43900 (96.8)	227 (0.5)	1.0	1.0	899 (2.0)	1.0	1.0	41150 (90.8)	223 (0.5)	1.0	1.0	842 (2.0)	1.0	1.0
	
	Possible	713 (1.6)	9 (1.3)	1.9	2.4* (1.0-3.8)	19 (2.7)	0.7	1.1 (0.5-1.7)	525 (1.2)	2 (0.4)	1.0	0.9 (0.2-1.6)	9 (1.7)	0.8	0.2 (0.1-0.3)
	
	Probable	728 (1.6)	8 (1.1)	2.0	2.3 (0.9-3.7)	11 (1.5)	1.0	1.0 (0.4-1.6)	3666 (8.1)	19 (0.5)	0.7	1.7 (0.9-2.5)	78 (2.1)	1.0	1.1 (0.6-1.6)

**Alkyl** **phenols**	Unlikely	43732 (96.5)	228 (0.5)	1.0	1.0	899 (2.1)	1.0	1.0	42959 (99.7)	228 (0.5)	1.0	1.0	894 (2.1)	1.0	1.0
	
	Possible	728 (1.6)	7 (1.0)	1.8	2.3 (0.9-3.7)	11 (1.5)	0.7	1.3 (0.4-2.2)	696 (1.5)	4 (0.6)	1.1	-	12 (1.7)	0.8	1.0 (0.5-1.5)
	
	Probable	881 (1.9)	9 (1.0)	2.0	2.3 (1.0-3.6)	19 (2.2)	1.0	1.1 (0.4-1.8)	1686 (3.7)	12 (0.7)	1.4	0.7 (0.3-1.1)	23 (1.4)	0.7	0.6 (0.3-0.9)

**Bis-phenols**	Unlikely	44371 (97.9)	237 (0.5)	1.0	1.0	912 (2.1)	1.0	1.0	44826 (98.9)	239 (0.5)	1.0	1.0	916 (2.0)	1.0	1.0
	
	Possible	147 (0.3)	-	-	-	4 (2.7)	1.3	1.9 (0.6-3.2)	297 (0.7)	4 (1.3)	2.5	1.0 (0.2-1.8)	4 (1.3)	0.7	1.2 (0.5-1.9)
	
	Probable	823 (1.8)	7 (0.9)	1.6	0.8 (0.2-1.4)	13 (1.6)	0.8	1.0 (0.4-1.6)	218 (0.5)	1 (0.5)	0.9	1.8 (0.8-2.8)	9 (4.1)	2.0*	1.8 (0.6-3.0)

**Heavy** **metals**	Unlikely	44725 (98.6)	240 (0.5)	1.0	1.0	917 (2.1)	1.0	1.0	44115 (97.3)	235 (0.5)	1.0	1.0	904 (2.0)	1.0	1.0
	
	Possible	220 (0.5)	1 (0.5)	0.8	2.2 (0.7-3.7)	6 (2.7)	1.4	1.0 (0.4-1.6)	440 (1.0)	5 (1.1)	2.2	2.2* (1.0-3.4)	6 (1.4)	0.7	1.2 (0.5-1.9)
	
	Probable	396 (0.9)	3 (0.8)	1.4	1.2 (0.4-2.0)	6 (1.5)	0.7	1.0 (0.3-1.7)	786 (1.7)	4 (0.5)	1.0	0.7 (0.2-1.2)	19 (2.4)	1.2	1.9* (1.1-2.7)

**Other** **compounds**	Unlikely	44667 (98.5)	236 (0.5)	1.0	1.0	921 (2.1)	1.0	1.0	43828 (96.7)	236 (0.5)	1.0	1.0	907 (2.1)	1.0	1.0
	
	Possible	674 (1.5)	8 (1.2)	2.2	2.8* (1.3-4.3)	8 (1.2)	0.6	1.0 (0.3-1.7)	1513 (3.3)	8 (0.5)	1.0	1.6 (0.8-2.4)	22 (1.5)	0.7	0.6 (0.3-0.9)

The Danish National Birth Cohort (1997-2009).

CIP = Cumulative Incidence Proportion per 100 boys.

CI = Confidence Interval.

HRa Hazard Ratio adjusted by age of the mother and the father; mothers' pre-pregnancy body mass index (kg/m^2^); earlier spontaneous abortion; parity; birth weight of boys; gestational age; oral contraceptives used; treatment of infertility; time taken to conceive; mother's alcohol consumption during pregnancy; binge drinking; maternal smoking during pregnancy; paternal smoking; gynecological disease; maternal vegetarian diet.

There was no difference in risk of cryptorchidism whether an orchiopexy was performed or not (Table 3).

**Table 3:** Hazard ratio (HR) for a cryptorchidism diagnosis with or without orchipexy among 45,341 Danish boys, according to parental occupational exposure to ECDs before and during pregnancy.

EDCs occupational exposures		Study Population (N = 45341) (%)	No orchiopexy (n = 426)			Orchiopexy (n = 576)		
			
			N (CIP)	HRc	HRa* (95% CI)	N (CIP)	HRc	HRa* (95% CI)
**Maternal** **exposure**	Unlikely	42474 (93.7)	378 (0.9)	1.0	1.0	498 (1.2)	1.0	1.0
	
	Possible	277 (0.6)	-	1.2	1.8 (0.5-3.1)	7 (2.5)	2.1	2.8 (0.7-4.9)
	
	Probable	2590 (5.7%)	16 (6.2)	0.9	0.9 (0.3-1.5)	30 (1.2)	1.0	0.7 (0.21.2)

**Paternal** **exposure**	Unlikely	38726 (85.4)	351 (0.9)	1.0	1.0	448 (1.2)	1.0	1.0
	
	Possible	410 (0.9)	3 (0.7)	0.8	0.5 (0.1-2.5)	4 (1.0)	0.8	0.6 (0.1-1.1)
	
	Probable	6205 (13.7)	40 (0.7)	1.0	1.2 (0.8-1.6)	83 (1.2)	1.1	1.3 (0.8-1.8)

CI = Confidence Interval.

CIP = Cumulative Incidence Proportion per 100 boys.

*HRa.- Hazard Ratio Adjusted by age of the mother and the father; mothers' pre-pregnancy body mass index (kg/m^2^); earlier spontaneous abortion; parity; birth weight of boys; gestational age; oral contraceptives used; treatment of infertility; time taken to conceive; mother's alcohol consumption during pregnancy; binge drinking; maternal smoking during pregnancy; paternal smoking; gynecological disease; maternal vegetarian diet.

## Discussion

We observed a modestly increased risk for hypospadias in relation to maternal occupational EDC exposure and paternal exposure to heavy metals while the risk of cryptorchidism was not increased. These results are not entirely consistent with the hypothesis that the two male disorders share EDC exposure as etiological factor. If occupational exposure to combined EDCs is increasing the risk of hypospadias and cryptorchidism, the risk should be elevated consistently according to several of the exposure subcategories, since all have expected EDC exposure in common but we don't see it in our results. Few and scattered associations do not indicate a strong association with endocrine disruption. It must of course be acknowledged that the various exposures may impact many biological pathways and that exposure prevalence and exposure levels within and across occupations may vary substantially.

The cumulative incidence of cryptorchidism of 2.2% in the studied sample is close to the rate of 1.9% observed in a prospective study of a subset of the DNBC [2], but less than the 3,2%, observed in a large representative sample of the Danish population [27]. This difference may be explained by shorter follow-up time in our study. Congenital cases of transient cryptorchidism may not be clinically recognized and reported to the register, as spontaneous descent is frequent during the first 3 months of life [2]. Cases are reported later if the condition persists, and the register-based cumulative incidence of cryptorchidism is increasing until 15 years of age [27]. Thus, the register-based cryptorchidism endpoint under-ascertains mild and transient cases of cryptorchidism compared to cohorts with clinical examinations, but the register does include both congenital and acquired cases [1]. With a main focus on persisting cryptorchidism, a lower sensitivity to transient cases will bias results if the conditions have different aetiology, or if the ascertainment rate varies by exposure level. We have no reason to suspect that the diagnosing or routine reporting of this condition is related to maternal occupation. There is evidence that different hormonal signals are involved in the abdominal and inguinal testicular descent [28], but this will hardly affect risk estimates, as testes retained in the abdomen only comprise a few percent of all cases of cryptorchidism [29].

The observed cumulative incidence rate of hypospadias of 0.6% does not include mild glandular and coronal cases and is therefore substantially lower than the prevalence of 4.7% that was reported in a study of newborn boys from the Copenhagen area [30]. Although the sensitivity of hospital register-based reporting is low, the specificity is probably high. As for cryptorchidism, the reported analyses of relative effect estimates are therefore unlikely to be biased, unless diagnosing and reporting is related to maternal occupation, which is quite unlikely. The registry ascertainment of the hypospadias diagnosis mainly addresses the more severe cases.

This study assesses maternal and paternal occupational exposure to potential EDCs by a job exposure matrix (JEM). The same occupational exposure criteria were applied to mothers and fathers. Thus, the JEM approach ignores possible gender-specific exposure profiles within occupations. Moreover, little is known about actual exposure levels and possible interactions among multiple endocrine disrupters [14]. In addition, the JEM neither distinguishes substances with different mechanisms or potency for endocrine disruption nor incorporates any possible changes in exposure over time. In short, a JEM based approach will yield underestimated associations if the exposure causes the diseases we study.

Occupational studies addressing the endocrine disruption hypothesis are few in spite of the fact that the occupational setting often confers much higher exposure levels than environmental sources, which probably also applies to EDCs. A limitation of our study is the lack of information on actual exposure levels at the workplace. Moreover, we do not know if jobs that according to the hygienic experts may to involve exposure to phthalates, for instance, are conferring higher exposures than the ubiquitous exposure of the general population to these compounds. Although the lack of (consistent) associations in this study indicates that EDCs defined without referring to specific mechanisms (estrogenic, anti-androgenic etc) have little importance in the occupational setting, such findings do not rule out that environmental exposures could be important. We did observe discordant effects of parental exposure to combined EDCs for hypospadias - maternal exposure was associated with elevated occurrence and paternal exposure was not. This would be expected for a causal maternal exposure, whereas confounding or indirect effects might present elevated risks for both maternal and paternal exposure.

The risk of hypospadias according to occupational exposure during pregnancy has been examined in five published studies that have applied the EDC job exposure matrix developed by van Tongeren et al in 2002 [14]. While no increased risk related to maternal exposure was found in a Dutch nationwide register-based study [31] and a Dutch case-referent study [32], three subsequent case-referent studies all reported increased risk related to exposure to one or more classes of endocrine disrupters [33], to heavy metals [16] or to phthalates [34]. A Spanish nested case-control study presented an increased risk of hypospadias or cryptorchidism (OR = 2.8; 95% CI: 1.1-7.2) in relation to the measured amount of xenobiotic estrogenic activity in blood [35]. The limited size of this study did not allow for separate analyses of the two urogenital malformations. Phthalates are of particular interest since several of the above studies reported elevated risk related to this class of anti-androgenic chemicals [15,16,32,33]. Our data were also indicative of an adverse effect of phthalates, but estimates were not statistically significant. Some phthalates and several phthalate metabolites inhibit androgen synthesis in the fetal Leydig cell at environmental exposure levels [36], and the compounds have been related to decreased anogenital distance [23,37], which is associated with hypospadias [38,39]. Hairdressers belong to the largest single occupational group with a probable exposure to phthalates and possibly other endocrine disrupting chemicals [14].

A study of concordance rates of cryptorchidism in twin brothers, full brothers and half brothers clearly point towards important etiologic factors in the intra-uterine environment provided by the mother [26]. Recently, persistent pesticides and brominated flame-retardants in human breast milk have been linked to cryptorchidism in a large prospective study [21,40]. A high cryptorchidism frecuency was indicated among sons of women working in greenhouses during pregnancy compared to an external reference group [40]. Furthermore, occupational exposure to pesticides was also associated with other adverse effects such as decreased penile length, testicular volume and serum concentrations of testosterone and inhibin B. Contrary to the above results, three large case-referent studies have failed to demonstrate consistent associations between blood concentrations of a number of biopersistent xenobiotics and occurrence of cryptorchidism [19,20,41], but these biomarkers have all been measured at time points outside pregnancy. We observed no excess risk of cryptorchidism by maternal occupational EDC exposure, but instead some inconsistent association on never observe effects of paternal exposures. Stratifying cryptorchidism cases by orchiopexy yielded comparable results in the two groups.

In conclusion, this study provides some but limited evidence that occupational exposure to endocrine disrupting chemicals in general increases the risk of hypospadias.

## Conclusions

The study provides some evidence that occupational exposure to possible endocrine disrupting chemicals during pregnancy increases the risk of hypospadias.

## Abbreviations

EDC: endocrine disrupting chemical; JEM: job exposure matrix; DNBC: Danish National Birth Cohort.

## Competing interests

The authors declare that they have no competing interests.

## Authors' contributions

JPB organized the study and contributed to the writing of the manuscript. MMMSV wrote the manuscript. LK contributed to the writing of the manuscript and contributed to the study design. JO provided expert advice on study organization and contributed to the writing of the manuscript. GUT, MSJ, CRH and AMT contributed by interpreting the results and helped to write the manuscript. MMMSV and ALLG did the data analysis. All the authors read and approved the final manuscript.

## References

[B1] Wohlfahrt-Veje C Boisen KA Boas M Damgaard IN Kai CM Schmidt IM Chellakooty M Suomi AM Toppari J Skakkebaek NE Main KM Acquired cryptorchidism is frequent in infancy and childhood Int J Androl 2009 32 423 428 10.1111/j.1365-2605.2008.00946.x 19515170

[B2] Boisen KA Kaleva M Main KM Virtanen HE Haavisto AM Schmidt IM Chellakooty M Damgaard IN Mau C Reunanen M Skakkebaek NE Toppari J Difference in prevalence of congenital cryptorchidism in infants between two Nordic countries Lancet 2004 363 1264 1269 10.1016/S0140-6736(04)15998-9 15094270

[B3] Cryptorchidism: a prospective study of 7500 consecutive male births, 1984-8. John Radcliffe Hospital Cryptorchidism Study Group Arch Dis Child 1992 67 892 899 10.1136/adc.67.7.892 1355643 PMC1793845

[B4] Berkowitz GS Lapinski RH Dolgin SE Gazella JG Bodian CA Holzman IR Prevalence and natural history of cryptorchidism Pediatrics 1993 92 44 49 8100060

[B5] Acerini CL Miles HL Dunger DB Ong KK Hughes IA The descriptive epidemiology of congenital and acquired cryptorchidism in a UK infant cohort Arch Dis Child 2009 94 868 872 10.1136/adc.2008.150219 19542061

[B6] Pierik FH Burdorf A Nijman JM de Muinck Keizer-Schrama SM Juttmann RE Weber R A high hypospadias rate in The Netherlands Hum Reprod 2002 17 1112 1115 10.1093/humrep/17.4.1112 11925415

[B7] Eurocat working group An assessment and analysis of existing surveillance data on hypospadias in UK and Europe 2003 University of Ulster

[B8] Aho M Koivisto AM Tammela TL Auvinen A Is the incidence of hypospadias increasing? Analysis of Finnish hospital discharge data 1970-1994 Environ Health Perspect 2000 108 463 465 10.1289/ehp.00108463 10811575 PMC1638043

[B9] Paulozzi LJ International trends in rates of hypospadias and cryptorchidism Environ Health Perspect 1999 107 297 302 10.1289/ehp.99107297 10090709 PMC1566511

[B10] Toppari J Kaleva M Virtanene HE Trends in the incidence of cryptorchisdism and hypospadias, and methodological limitations of registry-based data Human Reprod Update 2001 7 282 286 10.1093/humupd/7.3.282 11392374

[B11] Toppari J Environmental endocrine disrupters and disorders of sexual differentiation Semin Reprod Med 2002 20 305 312 10.1055/s-2002-35377 12428210

[B12] Skakkebaek NE Rajpert-De Meyts E Main KM Testicular dysgenesis syndrome: an increasingly common developmental disorder with environmental aspects Hum Reprod 2001 16 972 978 10.1093/humrep/16.5.972 11331648

[B13] Sharpe RM The 'oestrogen hypothesis'- where do we stand now? Int J Androl 2003 26 2 15 10.1046/j.1365-2605.2003.00367.x 12534932

[B14] van Tongeren M Nieuwenhuijsen MJ Gardiner K Armstrong B Vrijheid M Dolk H Botting B A job-exposure matrix for potential endocrine-disrupting chemicals developed for a study into the association between maternal occupational exposure and hypospadias Ann Occup Hyg 2002 46 465 477 10.1093/annhyg/mef053 12176761

[B15] Giordano F Abballe A De Felip E di Domenico A Ferro F Grammatico P Ingelido AM Marra V Marrocco G Vallasciani S Figà-Talamanca I Maternal exposures to endocrine disrupting chemicals and hypospadias in offspring Birth Defects Res A Clin Mol Teratol 2010 88 241 250 20196143 10.1002/bdra.20657

[B16] Nassar N Abeywardana P Barker A Bower Parental occupational exposure to potential endocrine disrupting chemicals and risk of hypospadias in infants Occup Environ Med 2009 67 585 589 10.1136/oem.2009.048272 19939854

[B17] Kurahashi N Kasai S Saijo Y Sata F Kishi R Exposure to endocrine disrupting chemicals and human health: a review of epidemiological studies focused on hypospadias and cryptorchidism Nippon Eiseigaku Zasshi 2005 60 15 22 15773293 10.1265/jjh.60.15

[B18] Longnecker MP Klebanoff MA Dunson DB Guo X Chen Z Zhou H Brock J Maternal serum level of the DDT metabolite DDE in relation to fetal loss in previous pregnancies Environ Res 2005 97 127 133 10.1016/S0013-9351(03)00108-7 15533328

[B19] McGlynn KA Guo X Graubard BI Brock JW Klebanoff MA Longnecker MP Maternal pregnancy levels of polychlorinated biphenyls and risk of hypospadias and cryptorchidism in male offspring Environ Health Perspect 2009 117 1472 1476 19750116 10.1289/ehp.0800389 PMC2737028

[B20] Small CM DeCaro JJ Terrell M Dominguez C Cameron LL Wirth J Marcus M Maternal exposure to a brominated flame retardant and genitourinary conditions in male offspring Environ Health Perspect 2009 117 1175 1179 19654930 10.1289/ehp.0800058 PMC2717147

[B21] Main KM Kiviranta H Virtanen HE Sundqvist E Tuomisto JT Tuomisto J Vartiainen T Skakkebaek NE Toppari J Flame retardants in placenta and breast milk and cryptorchidism in newborn boys Environ Health Perspect 2007 115 1519 1526 17938745 10.1289/ehp.9924 PMC2022640

[B22] Damgaard IN Jensen TK Nordic Cryptorchidism Study Group Petersen JH Skakkebaek NE Toppari J Main KM Risk factors for congenital cryptorchidism in a prospective birth cohort study PLoS One 2008 3 e3051 10.1371/journal.pone.0003051 18725961 PMC2516600

[B23] Main KM Mortensen GK Kaleva MM Boisen KA Damgaard IN Chellakooty M Schmidt IM Suomi AM Virtanen HE Petersen DV Andersson AM Toppari J Skakkebaek NE Human breast milk contamination with phthalates and alterations of endogenous reproductive hormones in infants three months of age Environ Health Perspect 2006 114 270 276 10.1289/ehp.8075 16451866 PMC1367843

[B24] Olsen J Melbye M Olsen SF Sørensen TI Aaby P Andersen AM Taxbøl D Hansen KD Juhl M Schow TB Sørensen HT Andresen J Mortensen EL Olesen AW Søndergaard C The Danish National Birth Cohort-its background, structure and aim Scand J Public Health 2001 29 300 307 10.1177/14034948010290040201 11775787

[B25] Nohr EA Frydenberg M Henriksen TB Olsen J Does low participation in cohort studies induce bias? Epidemiology 2006 17 413 418 10.1097/01.ede.0000220549.14177.60 16755269

[B26] DISCO-88 Statistics Denmark's Standard Classification of Occupations 1996 Copenhague Danmarks Statistik

[B27] Jensen MS Toft G Thulstrup AM Henriksen TB Olsen J Christensen K Bonde JP Cryptorchidism concordance in monozygotic and dizygotic twin brothers, full brothers, and half-brothers Fertil Steril 2010 93 124 129 10.1016/j.fertnstert.2008.09.041 19022430

[B28] Nef S Parada LF Hormones in male sexual development Genes Dev 2000 14 3075 3086 10.1101/gad.843800 11124800

[B29] Storgaard L Bonde JP Olsen J Male reproductive disorders in humans and prenatal indicators of estrogen exposure A review of published epidemiological studies Reprod Toxicol 2006 21 4 15 10.1016/j.reprotox.2005.05.006 16005180

[B30] Boisen K Chellakooty M Schmidt I Kai CM Damgaard IN Suomi AM Toppari J Skakkebaek NE Main KM Hypospadias in a cohort of 1072 Danish newborn boys: Prevalence and relationship to placental weight, anthropometrical measurements at birth, and reproductive hormone levels at 3 months of age J Clin Endocrinol Metab 2005 90 4041 4046 10.1210/jc.2005-0302 15870122

[B31] Vrijheid M Armstrong B Dolk H van Tongeren M Botting B Risk of hypospadias in relation to maternal occupational exposure to potential endocrine disrupting chemicals Occup Environ Med 2003 60 543 550 10.1136/oem.60.8.543 12883014 PMC1740604

[B32] Pierik FH Burdorf A Deddens JA Juttmann RE Weber RF Maternal and paternal risk factors for cryptorchidism and hypospadias: a case-control study in newborn boys Environ Health Perspect 2004 112 1570 1576 10.1289/ehp.7243 15531444 PMC1247623

[B33] Carbone P Giordano F Nori F Mantovani A Taruscio D Lauria L Figà-Talamanca I The possible role of endocrine disrupting chemicals in the aetiology of cryptorchidism and hypospadias: a population-based case-control study in rural Sicily Int J Androl 2007 30 3 13 10.1111/j.1365-2605.2006.00703.x 16824044

[B34] Ormond G Nieuwenhuijsen MJ Nelson P Toledano MB Iszatt N Geneletti S Elliott P Endocrine disruptors in the workplace, hair spray, folate supplementation, and risk of hypospadias: case-control study Environ Health Perspect 2009 117 303 307 19270804 10.1289/ehp.11933 PMC2649236

[B35] Fernandez MF Olmos B Granada A López-Espinosa MJ Molina-Molina JM Fernandez JM Cruz M Olea-Serrano F Human exposure to endocrine-disrupting chemicals and prenatal risk factors for cryptorchidism and hypospadias: a nested case-control study Environ Health Perspect 2007 115 8 14 10.1289/ehp.9351 18174944 PMC2174399

[B36] Mylchreest E Wallace DG Cattley RC Foster PM Dose-dependent alterations in androgen-regulated male reproductive development in rats exposed to Di(n-butyl) phthalate during late gestation Toxicol Sci 2000 55 143 151 10.1093/toxsci/55.1.143 10788569

[B37] Swan SH Main KM Liu F Stewart SL Kruse RL Calafat AM Mao CS Redmon JB Ternand CL Sullivan S Teague JL Study for Future Families Research Team Decrease in anogenital distance among male infants with prenatal phthalate exposure Environ Health Perspect 2005 113 1056 1061 10.1289/ehp.8100 16079079 PMC1280349

[B38] Mylchreest E Sar M Wallace DG Foster PM Fetal testosterone insufficiency and abnormal proliferation of Leydig cells and gonocytes in rats exposed to di(n-butyl) phthalate Reprod Toxicol 2002 16 19 28 10.1016/S0890-6238(01)00201-5 11934529

[B39] Lottrup G Andersson AM Leffers H Mortensen GK Toppari J Skakkebaek NE Main KM Possible impact of phthalates on infant reproductive health Int J Androl 2006 29 172 180 10.1111/j.1365-2605.2005.00642.x 16466537

[B40] Damgaard IN Skakkebaek NE Toppari J Virtanen HE Shen H Schramm KW Petersen JH Jensen TK Main KM Nordic Cryptorchidism Study Group Persistent pesticides in human breast milk and cryptorchidism Environ Health Perspect 2006 114 1133 1138 10.1289/ehp.8741 16835070 PMC1513324

[B41] Longnecker MP Klebanoff MA Brock JW Zhou H Gray KA Needham LL Wilcox AJ Maternal serum level of 1,1-dichloro-2,2-bis(p-chlorophenyl)ethylene and risk of cryptorchidism, hypospadias, and polythelia among male offspring Am J Epidemiol 2002 155 313 322 10.1093/aje/155.4.313 11836195

